# 2-Chloro-*N*′-(2-hy­droxy-3,5-diiodo­benzyl­idene)benzohydrazide

**DOI:** 10.1107/S1600536811007653

**Published:** 2011-03-09

**Authors:** Fei Wang, Da-Yong Liu, Hai-Bo Wang, Xian-Sheng Meng, Ting-Guo Kang

**Affiliations:** a School of Pharmacy, Liaoning University of Traditional Chinese Medicine, Shenyang 110032, People’s Republic of China; b Department of Chemistry and Chemical Engineering, Huanghuai University, Henan 463000, People’s Republic of China

## Abstract

In the title compound, C_14_H_9_ClI_2_N_2_O_2_, the dihedral angle between the benzene rings is 65.9 (2)° and an intra­molecular O—H⋯N hydrogen bond generates an *S*(6) ring. The mol­ecule has an *E* conformation about the C=N bond. In the crystal, mol­ecules are linked into *C*(4) chains propagating in [001] by N—H⋯O hydrogen bonds.

## Related literature

For background to hydrazone compounds and their biological properties, see: Kucukguzel *et al.* (2006 ▶); Khattab (2005 ▶); Karthikeyan *et al.* (2006 ▶); Okabe *et al.* (1993 ▶). For reference bond-length values, see: Allen *et al.* (1987 ▶). For related structures, see: Shan *et al.* (2008 ▶); Fun *et al.* (2008 ▶); Yang (2008 ▶); Ma *et al.* (2008 ▶); Diao *et al.* (2008*a* ▶,*b* ▶); Ejsmont *et al.* (2008 ▶).
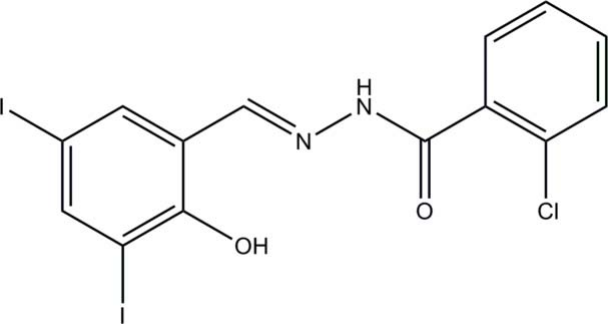


## Experimental

### 

#### Crystal data

C_14_H_9_ClI_2_N_2_O_2_
*M* *_r_* = 526.48
Monoclinic, 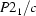

*a* = 14.311 (3) Å
*b* = 11.469 (2) Å
*c* = 9.736 (2) Å
β = 90.032 (2)°
*V* = 1598.0 (5) Å^3^
*Z* = 4
Mo *K*α radiation
μ = 4.11 mm^−1^
*T* = 298 K
0.18 × 0.17 × 0.17 mm

#### Data collection

Bruker SMART CCD diffractometer
Absorption correction: multi-scan (*SADABS*; Bruker, 2001 ▶) *T*_min_ = 0.525, *T*_max_ = 0.542
7381 measured reflections
3383 independent reflections
1747 reflections with *I* > 2σ(*I*)
*R*_int_ = 0.069

#### Refinement

*R*[*F* ^2^ > 2σ(*F* ^2^)] = 0.055
*wR*(*F* ^2^) = 0.112
*S* = 0.95
3383 reflections
194 parameters
1 restraint
H atoms treated by a mixture of independent and constrained refinement
Δρ_max_ = 0.93 e Å^−3^
Δρ_min_ = −0.80 e Å^−3^

### 

Data collection: *SMART* (Bruker, 2007 ▶); cell refinement: *SAINT* (Bruker, 2007 ▶); data reduction: *SAINT*; program(s) used to solve structure: *SHELXTL* (Sheldrick, 2008 ▶); program(s) used to refine structure: *SHELXTL*; molecular graphics: *SHELXTL*; software used to prepare material for publication: *SHELXTL*.

## Supplementary Material

Crystal structure: contains datablocks global, I. DOI: 10.1107/S1600536811007653/hb5809sup1.cif

Structure factors: contains datablocks I. DOI: 10.1107/S1600536811007653/hb5809Isup2.hkl

Additional supplementary materials:  crystallographic information; 3D view; checkCIF report

## Figures and Tables

**Table 1:** Hydrogen-bond geometry (Å, °)

*D*—H⋯*A*	*D*—H	H⋯*A*	*D*⋯*A*	*D*—H⋯*A*
O1—H1⋯N1	0.82	1.83	2.556 (8)	146
N2—H2⋯O2^i^	0.91 (4)	1.88 (2)	2.768 (8)	168 (8)

Symmetry code: (i) 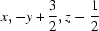.

**Fig. 1.**
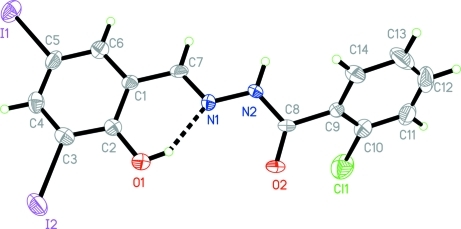
The molecular structure of the title compound with 30% probability displacement ellipsoids for non-H atoms. Intramolecular O—H···N hydrogen bond is drawn as a dashed line.

**Fig. 2.**
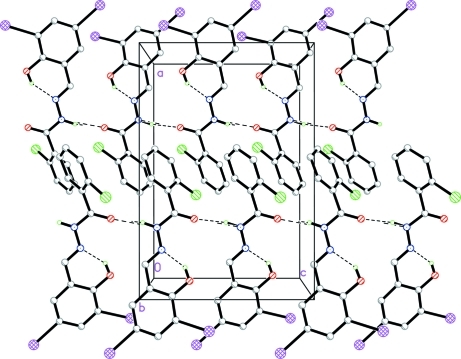
Molecular packing as viewed along the b axis. Hydrogen bonds are shown as dashed lines.

## supplementary crystallographic information

### Comment

Hydrazones have been attracted much attention for their excellent biological properties, especially for their potential pharmacological and antitumor properties (Kucukguzel *et al.*, 2006; Khattab *et al.*, 2005; Karthikeyan *et al.*, 2006; Okabe *et al.*, 1993). Recently, a large number of hydrazone derivatives have been prepared and structurally characterized (Shan *et al.*, 2008; Fun *et al.*, 2008; Yang, 2008; Ma *et al.*, 2008; Diao *et al.*, 2008*a*,*b*; Ejsmont *et al.*, 2008). In this paper, the title new hydrazone compound is reported.

The molecular structure of the title compound is shown in Fig. 1. The bond distances and angles are normal (Allen *et al.*, 1987). The dihedral angle between the two benzene rings is 65.9 (2)°. The molecule of the compound displays an *E* geometry about the C═N bond. The molecules are linked into chains along the *c* axis by intermolecular N—H···O hydrogen bonds (Fig. 2 and Table 1).

### Experimental

2-Hydroxy-3,5-diiodobenzaldehyde (1.0 mmol, 373.9 mg) was dissolved in methanol (50 ml), then 2-chlorobenzohydrazide (1.0 mmol, 170.6 mg) was added slowly into the solution, and the mixture was kept at reflux with continuous stirring for 2 h. After the solution had cooled to room temperature colourless powder crystals appeared. The powder crystals were filtered and washed with methanol for three times. Recrystallization from absolute methanol yielded colourless block-shaped single crystals of the title compound.

### Refinement

H2 was located in a difference Fourier map and refined isotropically, with N—H distance restrained to 0.90 (1) Å. Other H atoms were placed in calculated positions with O—H = 0.82 Å, C—H = 0.93 Å, and refined in riding mode with U_iso_(H) = 1.2U_eq_(C) and 1.5U_eq_(O).

### Crystal data

**Table:**

C_14_H_9_ClI_2_N_2_O_2_	*F*(000) = 984
*M**_r_* = 526.48	*D*_x_ = 2.188 Mg m^−^^3^
Monoclinic, *P*2_1_/*c*	Mo *K*α radiation, λ = 0.71073 Å
Hall symbol: -P 2ybc	Cell parameters from 989 reflections
*a* = 14.311 (3) Å	θ = 2.5–24.5°
*b* = 11.469 (2) Å	µ = 4.11 mm^−^^1^
*c* = 9.736 (2) Å	*T* = 298 K
β = 90.032 (2)°	Block, colourless
*V* = 1598.0 (5) Å^3^	0.18 × 0.17 × 0.17 mm
*Z* = 4	

### Data collection

**Table:**

Bruker SMART CCD diffractometer	3383 independent reflections
Radiation source: fine-focus sealed tube	1747 reflections with *I* > 2σ(*I*)
graphite	*R*_int_ = 0.069
ω scans	θ_max_ = 27.0°, θ_min_ = 2.7°
Absorption correction: multi-scan (*SADABS*; Bruker, 2001)	*h* = −18→13
*T*_min_ = 0.525, *T*_max_ = 0.542	*k* = −14→9
7381 measured reflections	*l* = −12→12

### Refinement

**Table:**

Refinement on *F*^2^	Primary atom site location: structure-invariant direct methods
Least-squares matrix: full	Secondary atom site location: difference Fourier map
*R*[*F*^2^ > 2σ(*F*^2^)] = 0.055	Hydrogen site location: inferred from neighbouring sites
*wR*(*F*^2^) = 0.112	H atoms treated by a mixture of independent and constrained refinement
*S* = 0.95	*w* = 1/[σ^2^(*F*_o_^2^) + (0.0353*P*)^2^] where *P* = (*F*_o_^2^ + 2*F*_c_^2^)/3
3383 reflections	(Δ/σ)_max_ < 0.001
194 parameters	Δρ_max_ = 0.93 e Å^−^^3^
1 restraint	Δρ_min_ = −0.80 e Å^−^^3^

### Special details

**Table:**

Geometry. All esds (except the esd in the dihedral angle between two l.s. planes) are estimated using the full covariance matrix. The cell esds are taken into account individually in the estimation of esds in distances, angles and torsion angles; correlations between esds in cell parameters are only used when they are defined by crystal symmetry. An approximate (isotropic) treatment of cell esds is used for estimating esds involving l.s. planes.
Refinement. Refinement of F^2^ against ALL reflections. The weighted R-factor wR and goodness of fit S are based on F^2^, conventional R-factors R are based on F, with F set to zero for negative F^2^. The threshold expression of F^2^ > 2sigma(F^2^) is used only for calculating R-factors(gt) etc. and is not relevant to the choice of reflections for refinement. R-factors based on F^2^ are statistically about twice as large as those based on F, and R- factors based on ALL data will be even larger.

### Fractional atomic coordinates and isotropic or equivalent isotropic displacement parameters (Å^2^)

**Table:**

	*x*	*y*	*z*	*U*_iso_*/*U*_eq_	
I1	−0.22657 (4)	0.87447 (6)	−0.18612 (7)	0.0664 (2)	
I2	−0.12679 (4)	0.97420 (6)	0.39763 (7)	0.0689 (2)	
Cl1	0.39839 (19)	0.5079 (3)	0.3068 (3)	0.0908 (9)	
N1	0.1820 (4)	0.8044 (6)	0.1129 (6)	0.0385 (16)	
N2	0.2702 (4)	0.7650 (6)	0.0825 (6)	0.0402 (17)	
O1	0.0634 (3)	0.8886 (5)	0.2821 (5)	0.0482 (14)	
H1	0.1148	0.8671	0.2551	0.072*	
O2	0.3053 (4)	0.7548 (6)	0.3039 (6)	0.077 (2)	
C1	0.0291 (5)	0.8487 (6)	0.0472 (8)	0.0343 (19)	
C2	0.0025 (5)	0.8833 (6)	0.1793 (8)	0.0367 (19)	
C3	−0.0897 (5)	0.9172 (7)	0.2027 (8)	0.043 (2)	
C4	−0.1538 (5)	0.9133 (7)	0.0998 (10)	0.050 (2)	
H4	−0.2153	0.9350	0.1168	0.060*	
C5	−0.1283 (5)	0.8778 (8)	−0.0279 (9)	0.051 (2)	
C6	−0.0383 (5)	0.8446 (6)	−0.0562 (8)	0.044 (2)	
H6	−0.0222	0.8196	−0.1439	0.053*	
C7	0.1229 (5)	0.8119 (7)	0.0171 (8)	0.0378 (19)	
H7	0.1400	0.7937	−0.0725	0.045*	
C8	0.3274 (5)	0.7392 (8)	0.1856 (8)	0.046 (2)	
C9	0.4215 (5)	0.6948 (8)	0.1424 (8)	0.044 (2)	
C10	0.4584 (6)	0.5931 (8)	0.1939 (9)	0.055 (2)	
C11	0.5447 (7)	0.5530 (10)	0.1535 (11)	0.074 (3)	
H11	0.5699	0.4843	0.1882	0.088*	
C12	0.5921 (7)	0.6211 (15)	0.0577 (14)	0.102 (6)	
H12	0.6510	0.5968	0.0293	0.122*	
C13	0.5576 (8)	0.7187 (12)	0.0048 (12)	0.090 (4)	
H13	0.5915	0.7607	−0.0599	0.108*	
C14	0.4710 (6)	0.7572 (9)	0.0471 (9)	0.068 (3)	
H14	0.4462	0.8255	0.0109	0.082*	
H2	0.290 (5)	0.755 (8)	−0.005 (3)	0.080*	

### Atomic displacement parameters (Å^2^)

**Table:**

	*U*^11^	*U*^22^	*U*^33^	*U*^12^	*U*^13^	*U*^23^
I1	0.0465 (4)	0.0791 (5)	0.0737 (5)	−0.0086 (3)	−0.0190 (3)	0.0115 (4)
I2	0.0617 (4)	0.0786 (5)	0.0663 (5)	0.0136 (3)	0.0127 (3)	−0.0186 (4)
Cl1	0.0839 (19)	0.089 (2)	0.099 (2)	−0.0011 (15)	−0.0081 (16)	0.0317 (18)
N1	0.028 (4)	0.052 (5)	0.036 (4)	0.004 (3)	−0.002 (3)	−0.002 (3)
N2	0.034 (4)	0.063 (5)	0.024 (4)	0.008 (3)	0.004 (3)	−0.005 (4)
O1	0.051 (3)	0.058 (4)	0.036 (3)	0.008 (3)	0.000 (3)	−0.002 (3)
O2	0.068 (4)	0.143 (7)	0.020 (3)	0.042 (4)	0.003 (3)	0.003 (4)
C1	0.041 (5)	0.031 (5)	0.031 (5)	−0.001 (3)	0.002 (3)	0.008 (4)
C2	0.035 (4)	0.035 (5)	0.040 (5)	−0.011 (4)	0.004 (3)	0.008 (4)
C3	0.051 (5)	0.034 (5)	0.044 (6)	−0.007 (4)	0.010 (4)	0.002 (4)
C4	0.034 (5)	0.048 (6)	0.067 (7)	0.000 (4)	0.004 (4)	0.006 (5)
C5	0.037 (5)	0.061 (6)	0.055 (6)	0.000 (4)	−0.011 (4)	0.014 (5)
C6	0.049 (5)	0.038 (5)	0.045 (5)	−0.003 (4)	−0.006 (4)	−0.008 (4)
C7	0.051 (5)	0.038 (5)	0.025 (5)	0.002 (4)	0.003 (4)	0.009 (4)
C8	0.047 (5)	0.070 (7)	0.020 (5)	0.007 (4)	0.002 (4)	0.000 (4)
C9	0.030 (4)	0.072 (7)	0.029 (5)	0.000 (4)	−0.002 (3)	−0.008 (5)
C10	0.048 (6)	0.066 (7)	0.052 (6)	−0.001 (5)	−0.007 (4)	−0.003 (5)
C11	0.054 (7)	0.100 (10)	0.067 (8)	0.026 (6)	−0.018 (5)	−0.025 (7)
C12	0.039 (6)	0.182 (16)	0.083 (10)	0.015 (8)	−0.013 (6)	−0.079 (11)
C13	0.066 (8)	0.141 (13)	0.063 (8)	−0.034 (8)	0.029 (6)	−0.016 (8)
C14	0.050 (6)	0.112 (9)	0.043 (6)	−0.006 (6)	0.010 (4)	0.007 (6)

### Geometric parameters (Å, °)

**Table:**

I1—C5	2.086 (7)	C4—H4	0.9300
I2—C3	2.076 (8)	C5—C6	1.372 (10)
Cl1—C10	1.703 (9)	C6—H6	0.9300
N1—C7	1.261 (8)	C7—H7	0.9300
N1—N2	1.373 (7)	C8—C9	1.501 (10)
N2—C8	1.328 (9)	C9—C14	1.369 (11)
N2—H2	0.91 (4)	C9—C10	1.374 (12)
O1—C2	1.328 (8)	C10—C11	1.376 (11)
O1—H1	0.8200	C11—C12	1.393 (16)
O2—C8	1.208 (9)	C11—H11	0.9300
C1—C6	1.395 (10)	C12—C13	1.328 (16)
C1—C2	1.398 (10)	C12—H12	0.9300
C1—C7	1.438 (9)	C13—C14	1.379 (13)
C2—C3	1.396 (10)	C13—H13	0.9300
C3—C4	1.358 (10)	C14—H14	0.9300
C4—C5	1.358 (11)		
			
C7—N1—N2	118.6 (6)	N1—C7—H7	120.2
C8—N2—N1	118.5 (6)	C1—C7—H7	120.2
C8—N2—H2	119 (5)	O2—C8—N2	121.8 (7)
N1—N2—H2	122 (5)	O2—C8—C9	123.5 (7)
C2—O1—H1	109.5	N2—C8—C9	114.6 (7)
C6—C1—C2	119.0 (7)	C14—C9—C10	119.5 (8)
C6—C1—C7	119.2 (7)	C14—C9—C8	118.5 (8)
C2—C1—C7	121.7 (6)	C10—C9—C8	122.0 (8)
O1—C2—C3	118.9 (7)	C9—C10—C11	121.6 (9)
O1—C2—C1	121.8 (7)	C9—C10—Cl1	121.9 (7)
C3—C2—C1	119.2 (7)	C11—C10—Cl1	116.5 (8)
C4—C3—C2	120.5 (8)	C10—C11—C12	116.2 (10)
C4—C3—I2	120.8 (6)	C10—C11—H11	121.9
C2—C3—I2	118.6 (6)	C12—C11—H11	121.9
C5—C4—C3	120.3 (8)	C13—C12—C11	123.5 (12)
C5—C4—H4	119.9	C13—C12—H12	118.3
C3—C4—H4	119.9	C11—C12—H12	118.3
C4—C5—C6	121.3 (7)	C12—C13—C14	119.2 (12)
C4—C5—I1	120.0 (6)	C12—C13—H13	120.4
C6—C5—I1	118.7 (7)	C14—C13—H13	120.4
C5—C6—C1	119.7 (8)	C9—C14—C13	120.0 (10)
C5—C6—H6	120.2	C9—C14—H14	120.0
C1—C6—H6	120.2	C13—C14—H14	120.0
N1—C7—C1	119.7 (7)		

### Hydrogen-bond geometry (Å, °)

**Table:**

*D*—H···*A*	*D*—H	H···*A*	*D*···*A*	*D*—H···*A*
O1—H1···N1	0.82	1.83	2.556 (8)	146
N2—H2···O2^i^	0.91 (4)	1.88 (2)	2.768 (8)	168 (8)

Symmetry codes: (i) *x*, −*y*+3/2, *z*−1/2.
